# Contraceptive implants: providing better choice to meet growing family planning demand

**DOI:** 10.9745/GHSP-D-12-00003

**Published:** 2013-03-21

**Authors:** Roy Jacobstein, Harriet Stanley

**Affiliations:** a EngenderHealth, New York, NY, USA

## Abstract

Contraceptive implants are extremely effective, long acting, and suitable for nearly all women—to delay, space, or limit pregnancies—and they are increasingly popular. Now, markedly reduced prices and innovative service delivery models using dedicated non-physician service providers offer a historic opportunity to help satisfy women's growing need for family planning.

Contraceptive implants offer immense potential to meet the need for family planning. More than 220 million women in developing countries currently have an unmet need for modern contraception, mainly in South Asia and sub-Saharan Africa.^1^ Many other women are using less effective “resupply” methods—short-acting methods that require users to continually replenish their supplies of the contraceptive—because highly effective, more convenient methods such as implants are not easily accessible. In all countries, access is lower among poorer, less educated, rural, and younger women.^1-2^ From January 1, 2009, to December 31, 2012, more than 9 million implants valued at over US$190 million have been shipped to developing countries—87% to sub-Saharan Africa.^3^ The magnitude of commodity provision is likely to increase markedly, due to major price reductions.

What are implants? Why do they offer so much promise? What challenges must programs address to make them even more widely accessible and used?

## THE PROMISE OF IMPLANTS

### What Women Like About Implants

Implants are a long-acting, reversible form of progestin-only contraception that release an ultra-low amount of progestin continuously into the bloodstream. Currently, 3 implants are available: *Implanon*^®^, *Jadelle*^®^, and *Sino-implant II*^®^ (see Table). Women who use implants find them to be very convenient—they are effective immediately and offer up to 3 to 5 years of extremely reliable contraceptive protection upon one client action. Only a brief, very minor surgical procedure under local anesthesia is needed to place 1 or 2 matchstick-sized plastic rods beneath the skin of the inner upper arm.^4-5^ Some women also like that pelvic exams and laboratory tests are not required and that implants can be used discreetly. Furthermore, implants do not interfere with sexual intercourse, and return to fertility upon removal is not delayed or negatively affected.

**Table.** Key Characteristics of the 3 Available Contraceptive Implants

	Implanon®	Jadelle®	Sino-implant II®
Manufacturer	Merck	Bayer HealthCare	Shanghai Dahua
Active ingredient and amount	68 mg etonogestrel	150 mg levonorgestrel	150 mg levonorgestrel
Labeled duration of effective use	3 years	5 years	4 years
No. of rods	1	2	2
Approximate insertion and removal times	Insertion: 1 min	Insertion: 2 min	Insertion: 2 min
	Removal: 2–3 min	Removal: 5 min	Removal: 5 min
Cost of implant (US$)	$16.50^a^	$8.50	∼ $8.00

a The cost of *Implanon* may be lowered in the future to be comparable with that of *Jadelle*.

*Source: Modified from a table prepared by FHI 360, the RESPOND Project, and USAID*.

### Unmatched Effectiveness

Effectiveness is a key feature for women and couples using contraception to avoid unwanted pregnancy, but in our experience, even family planning professionals do not always fully realize just how effective implants are: Only 1 unintended pregnancy occurs among every 2,000 implant users in the first year of use.^6^ In contrast, failure rates in the first year of typical use of the commonly used resupply methods are considerably higher: 180 unintended pregnancies per 1,000 users of male condoms, 90 unintended pregnancies per 1,000 users of pills, and 60 unintended pregnancies per 1,000 users of the progestin-only injectable *Depo-Provera^®^*.^6^ Thus, implants are 120 times more effective than the injectable, 180 times more effective than the pill, and 360 times more effective than the condom.

### Suitable for All Reproductive Intentions and Nearly All Women

Implants are an excellent choice to achieve any reproductive intention—to delay a first pregnancy, space a subsequent birth, or end childbearing. According to the World Health Organization's (WHO's) *Medical Eligibility Criteria for Contraceptive Use*^7^ and *Family Planning: A Global Handbook for Providers*,^4^ implants are safe and suitable for nearly all women, including women who are of any age (including adolescents), have never been pregnant or have never had children, are living with HIV, have just had an abortion, or are breastfeeding.

Implants are safe and suitable for nearly all women.

With only one action, women who use implants can be almost certain not to have an unintended pregnancy for up to 3 to 5 years.

Implants are over 100 times more effective than injectables and pills in typical use, and 360 times more effective than condoms.

Recommendations among normative bodies differ about the suitability of implants use by breastfeeding women during the first 6 weeks after childbirth, however. WHO guidance states that the risks outweigh benefits during this period.^7^ The U.S. Centers for Disease Control and Prevention (CDC) advises that the benefits outweigh risks during the first 4 weeks and places no restrictions on use after 4 weeks.^8^ The U.K.'s Royal College of Obstetricians and Gynaecologists places no restrictions on use of implants by breastfeeding women at *any* time.^9^ Immediate postpartum provision of implants would offer expanded programmatic opportunity, as women are increasingly receiving safe delivery services and there is almost universal interest among postpartum women in avoiding a pregnancy for at least 2 years.^10^

Implants also offer great promise for helping to meet the needs of younger women, who often face many barriers in accessing effective modern contraception. When implants were made available to young Kenyan women ages 18–24 seeking family planning, 24% selected the method.^11^ The American College of Obstetricians and Gynecologists recommends that providers encourage adolescents ages 15–19 seeking contraception to consider implants and intrauterine devices (IUDs) as “the best reversible methods for preventing unintended pregnancy, rapid repeat pregnancy, and abortion in young women.”^12^

### Rising Popularity

Although modern contraceptive use lags in sub-Saharan Africa, where only 1 in 6 married women uses it, contraceptive use has recently increased substantially in a number of Eastern and Southern African countries.^13^ While this has been mainly due to increased use of injectables, implants use has also increased notably over a short time span in countries such as Ethiopia, Malawi, Rwanda, and Tanzania (see Box and Figure). For example, 1 in every 7 women using modern contraception in Rwanda currently relies on an implant, compared with less than 1 in 25 in 2005.^14^ These trends suggest that wider availability of implants could lead to much greater use in other African countries and elsewhere where implants currently cannot be accessed widely or easily. High rates of user satisfaction (79%) and continuation (around 84% at 1 year of use) further support this likelihood.^6^^,^^17^

**FIGURE.**
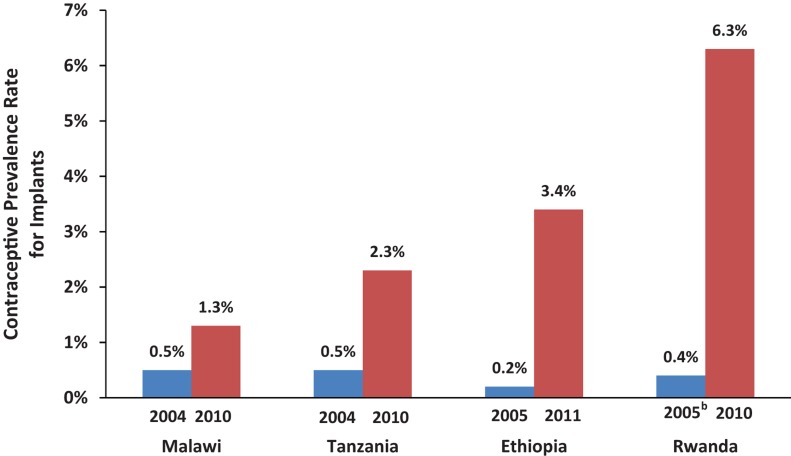
Increased Use of Implants in Malawi, Tanzania, Ethiopia, and Rwanda, 2004–2011^a^ ^a^ All data are for currently married women ages 15–49. ^b^ The 2005 Rwanda survey grouped use of implants with “other modern methods.” Data from the Demographic and Health Surveys.^14^

**Box. Implants Use on the Upswing in Eastern and Southern African Countries**

Ethiopia, Malawi, Rwanda, and Tanzania have recently achieved notable increases in their modern method contraceptive prevalence rates (CPR), including for implants. As seen in the Figure, in only 5 to 6 years, implants use doubled in Malawi, quadrupled in Tanzania, and rose more than 15-fold in Rwanda and 17-fold in Ethiopia.^14^ Implants have become the second most popular method in Ethiopia and the third most popular method in Rwanda. One of every 7 married women using modern contraception in Rwanda and 1 in every 8 in Ethiopia relies on an implant for her contraceptive protection. The CPR for implants in Rwanda is 6.3% among currently married women, 5.9% among sexually active unmarried women, and 6.4% among rural women. These are the highest rates in sub-Saharan Africa and among the highest in the world.

What accounts for this success? Among the most important factors have been:

- An **enabling environment**, with strong policy commitment from the highest levels downward, as manifested most recently by the Prime Ministers of Ethiopia and Rwanda at the London Summit,^15^ and supportive service policies that encourage task sharing and task shifting;
- On the **supply side**, training to ensure widespread insertion and removal skills and substantial donor support for purchase of commodities (3.7 million implants valued at US$72 million were purchased for these 4 African countries between 2009 and 2012)^16^; and
- On the **demand side**, a marked rise in implants knowledge, stimulated by communication activities in programs as well as by diffusion of knowledge among women themselves. In Ethiopia, knowledge of implants among married women ages 15–49 increased to 69% in 2011 from only 20% in 2005, and knowledge was even higher among sexually active, unmarried women (82%).^14^ In Rwanda, where only half of married women knew of implants in 2005, such knowledge became universal (97%) by 2010.^14^

### Increasingly Affordable and Available

Prospects for increased availability and use were greatly enhanced when Bayer HealthCare recently announced that it would cut the public-sector price of its contraceptive implant *Jadelle* in half, as a result of volume guarantees from international donor partners.^18^ Beginning in January 2013, *Jadelle* will cost US$8.50 per set. The partnership initiative aims to make 27 million implants available to the public sector and non-commercial private sector in up to 69 low-income countries from 2013 to 2018. This is likely to be a signal milestone on the long road toward wider use of implants. The commodity cost of implants—once as high as US$23.80 per set—has been a major impediment to their wider availability. (In comparison, the public-sector commodity cost of a Copper-T IUD ranges from US$0.36 to $0.48.^1^) Having 3 implants in the market appears to have helped induce these lower commodity prices, and hopefully prices will continue to fall.

## WHAT PROGRAMS CAN DO

### Ensure a Client-Centered Approach

A knowledgeable, empowered client is central to the provision and receipt of quality family planning services.^19^ This entails that programs provide and ensure:

- **Informed choice** from among a wide range of contraceptive options. (This has long been the bedrock principle of organized family planning programs.)
- Thoughtful **counseling** to help clients select a method, discuss its characteristics, and dispel myths and misconceptions (for example, that implants might migrate within the body). Counseling should also make clear that clients do not need to use an implant for its full length of labeled use in order to receive it.
- **Anticipatory guidance** regarding common side effects of implants, especially about bleeding disturbances and their acceptability to the client within her sociocultural context. This is particularly important, as changes in menstrual patterns—irregular, infrequent, or no bleeding—while not harmful are expected.^20^ The specific pattern in any given woman cannot be predicted with certainty, however.
- Capable and reassuring **management of side effects**, especially of bleeding changes, is thus often the difference between satisfaction and discontinuation. Side effects and health concerns are the main reason why women discontinue hormonal methods,^21^ and bleeding unpredictability is a chief reason why they discontinue implants.^20^
- Regular and reliable access to prompt **removal** services.
- Adequate **follow-up**, including making it clear that although the client does not *need* to return, she can and should return at any time, whether for advice, reassurance, treatment of side effects, or removal.

Counseling about, and management of, bleeding side effects are key to helping women use implants successfully.

### Appreciate and Nurture Providers

In addition to focusing on the client, programs must be attentive to the perspectives, needs, and workloads of providers.^22-23^ Without adequate availability and distribution of skilled, motivated, and enabled providers, there can be little provision of implants.^24^ In other words, “No provider, no program.” Fortunately for resource-constrained programs, many categories of health care providers—not only doctors but also nurses, midwives, auxiliary nurses, auxiliary nurse-midwives, and clinical officers—are capable, once trained, of safely providing implants.^25^ Such “task shifting” or “task sharing” among health cadres is an accepted policy and programmatic reality.^25-26^ Ethiopia has even launched a program to train and enable 15,000 rural community health extension workers (CHEWs) to insert *Implanon*,^27^ whose one rod can be inserted easily in 1 to 2 minutes. (Removals are handled by referral to higher-level cadres.)

Many cadres of health care providers can provide implants safely and effectively.

### Ensure Access to Removal Services

Programs also need to ensure **routine, regular, and reliable removal services** for clients, beginning by planning for them at the outset of service expansion efforts. Failure to provide reliable and ready access to removal services could easily tarnish the method's image and undermine an entire family planning program.

### Consider Use of Dedicated Providers and Mobile Services

A number of countries have successfully followed innovative public-private service delivery models that entail the use of “dedicated providers.” These providers focus primarily on delivering underutilized clinical contraceptive methods, including implants. (A wider range of method choices is generally available at the service site.) The service models have also typically entailed task shifting and provision of free services, either in static public-sector service sites or through mobile outreach.

In Zambia, 18 retired midwives were placed at high-volume, public-sector facilities solely to provide long-acting and reversible contraceptives (LARCs). These dedicated providers inserted more than 22,000 implants and 11,000 IUDs in 14 months and reached younger and lower-parity women.^28^ In Tanzania, a policy shift allowed nurses, as well as physicians, to provide implants. Subsequently, insertions more than doubled, from around 10,000 per quarter in 2007 to more than 20,000 per quarter in 2009,^29^ and nurses became the main providers of implants. In Malawi, where use of dedicated, non-physician providers and mobile services contributed substantially to wide use of female sterilization (prevalence of 10% among married women in 2010),^30^ implants provision through the same service modalities and providers also rose (see Box).

### Other Program and Health System Considerations

The following programmatic subsystems must be in place and functional to ensure that quality implant insertion and removal services can be regularly and reliably provided^19^^,^^24^:

- Commodity logistics and supplies
- Supervision and management
- Infection prevention and quality control
- Pre-service and in-service training (for all cadres who provide implants)
- Health communication, demand creation, and marketing
- Client follow-up

The environment also must be enabling and supportive, with:

- Strong political commitment
- Adequate and well-deployed financial and human resources
- Service delivery policies, guidelines, and standards that permit task shifting and task sharing to allow other skilled cadres besides doctors to provide implants
- No restrictions on access because of age, parity, marital status, HIV status, or socioeconomic status
- Widespread gender equity

Innovative models utilizing dedicated non-physician providers have increased availability and use of implants.

Finally, recurrent costs for infrastructure and staff must be met. Despite these costs, the overall cost of implants per couple-year of protection (CYP) is comparable to or less than that of injectables or oral contraceptives, and cost effectiveness rises with longer use.^31-33^

The overall cost of implants per CYP is comparable to or less than that of injectables or pills.

## WHAT IS AT STAKE

A woman in sub-Saharan Africa faces a 1 in 39 lifetime risk of maternal death, and a woman in South Asia has a 1 in 150 lifetime risk.^34^ In contrast, the lifetime risk of maternal death in industrialized countries is 1 in 4,700. Nearly all maternal deaths—99%—occur in low-resource countries,^35^ and for every instance of maternal mortality, 20 instances of serious morbidity (such as obstetric fistula) occur.^36^ Risk of morbidity and mortality is higher among poorer women, who have less access to modern contraception including implants. Access is also more constrained for young women, among whom 44% of all unintended pregnancies in sub-Saharan Africa occur.^10^

Meeting unmet need for contraception could prevent more than 100,000 maternal deaths each year.

Satisfying unmet need for contraception could reduce maternal mortality by 29%, preventing more than 100,000 maternal deaths each year.^34^ If only 1 of 5 sub-Saharan African women now using pills or injectables (that is, other, less effective hormonal contraception) were to switch to an implant, more than 1.8 million unintended pregnancies would be averted in 5 years, resulting in almost 600,000 fewer abortions and 10,000 fewer maternal deaths.^37^ If even more women were to switch, or if women not currently using contraception were to access and use implants, even greater individual and public health would accrue. Meeting the need for effective modern contraception, including much wider provision of contraceptive implants to women who would want them, is not only a family planning and health issue—it is a matter of social justice and an equity imperative.

## OUR CHALLENGE

The 2012 London Summit on Family Planning, attended by more than 150 leaders and representatives of governments and civil society, endorsed an ambitious goal of providing family planning to an additional 120 million women.^38^ Widespread provision of implants in a quality manner offers a substantial way to help achieve this goal, if the global health community can rise to the challenge. We must do it right, and do it now.
