# Corrigendum: Jacobstein et al., Contraceptive implants: providing better choice to meet growing family planning demand

**DOI:** 10.9745/GHSP-D-13-00099

**Published:** 2013-08-14

**Authors:** Roy Jacobstein, Harriett Stanley

**Affiliations:** a EngenderHealth, New York, NY, USA

In the article “Contraceptive implants: providing better choice to meet growing family planning demand” by Roy Jacobstein and Harriet Stanley, which appeared in the March 2013 issue (Volume 1, Issue 1), the second sentence in the box on page 14 incorrectly stated implants use rose 31-fold in Rwanda. This has been corrected to “more than 15-fold.”

In addition, on page 11, in the first sentence of the first paragraph under “What Women Like About Implants,” the term ultra-low “dose” was changed to ultra-low “amount,” to avoid the incorrect implication that implants deliver hormones into a woman's body in a single dose. In the third sentence, the missing word “protection” was added after “contraceptive” (…offer up to 3 to 5 years of extremely reliable contraceptive protection…).

The article has been corrected accordingly.

